# Using fluorescent promoter-reporters to study sugar utilization control in *Bifidobacterium longum* NCC 2705

**DOI:** 10.1038/s41598-022-14638-4

**Published:** 2022-06-21

**Authors:** S. Duboux, J. A. Muller, F. De Franceschi, A. Mercenier, M. Kleerebezem

**Affiliations:** 1 Nestlé Research, Lausanne, Switzerland; 2 Host-Microbe Interactomics Group, Wageningen University and Research, De Elst 1, 6708WD Wageningen, The Netherlands

**Keywords:** Bacterial physiology, Bacterial techniques and applications

## Abstract

Bifidobacteria are amongst the first bacteria to colonize the human gastro-intestinal system and have been proposed to play a crucial role in the development of the infant gut since their absence is correlated to the development of diseases later in life. Bifidobacteria have the capacity to metabolize a diverse range of (complex) carbohydrates, reflecting their adaptation to the lower gastro-intestinal tract. Detailed understanding of carbohydrate metabolism regulation in this genus is of prime importance and availability of additional genetic tools easing such studies would be beneficial. To develop a fluorescent protein-based reporter system that can be used in *B. longum* NCC 2705, we first selected the most promising fluorescent protein out of the seven we tested (i.e., mCherry). This reporter protein was then used to study the carbohydrate mediated activation of P_*Bl1518*_ and P_*Bl1694*_, two promoters respectively predicted to be controlled by the transcriptional factors AraQ and AraU, previously suggested to regulate arabinose utilization and proposed to also act as global transcriptional regulators in bifidobacteria. We confirmed that in *B. longum* NCC 2705 the AraQ controlled promoter (P_*Bl1518*_) is induced strongly by arabinose and established that the AraU controlled promoter (P_*Bl1694*_) was mostly induced by the hexoses galactose and fructose. Combining the mCherry reporter system with flow cytometry, we established that NCC 2705 is able to co-metabolize arabinose and glucose while galactose was only consumed after glucose exhaustion, thus illustrating the complexity of different carbohydrate consumption patterns and their specific regulation in this strain.

## Introduction

*Bifidobacterium* is an important genus within the human intestinal microbiota. Bifidobacterial species are among the first to establish in the neonate human gastrointestinal tract (GIT) and are predominant in the breast-fed newborn until the age of 4 months^1^. Their presence in early life has been proposed to play a critical role in the maturation of the newborn’s immune system as several studies have linked low abundance of members of this genus in the infant gut to later in life immune disorders such as atopy^2^ or asthma^3^. Early-life *Bifidobacterium* dominance has also been proposed to be linked with reduced risks of obesity^4^ or acquisition of antimicrobial resistances^5^ later in life.

The genome of bifidobacteria encodes an extensive enzyme repertoire that supports their capacity to effectively breakdown and metabolize diverse carbohydrate substrates^6^. The diversity of carbohydrates that can be metabolized by bifidobacteria is well reflected within the *B. longum* subspecies. Strains of *B. longum* subsp. *infantis* have the particular ability to hydrolyze several of the indigestible human milk oligosaccharides (HMOs) and to metabolize the derived galactose and fucose, explaining their abundance in the intestine of breast-fed infants^7^. Strains of *B. longum* subsp. *longum* can also metabolize carbohydrates found in human milk such as lactose^8^ or Lacto-N-Tetraose (LNT), but they appear to be particularly well equipped to import and consume food fibers derived carbohydrates, as reflected by the genome of *B. longum* subsp. *longum* NCC 2705 strain^9^. Bifidobacteria can be stimulated by the consumption of arabinose-containing cereal fibers^10^. This type of high molecular weight fiber (i.e., arabinogalactans) is first degraded by specific members of the microbiota community such as *Bacteroides caccae,* . producing smaller arabinose containing oligosaccharides that can further be consumed by secondary degraders as demonstrated for *B. longum* NCC 2705^11^. In *Bifidobacterium*, all carbohydrates are theoretically processed through the relatively unique central carbon metabolism that includes the so-called “bifid-shunt”, which involves the bifunctional xylulose 5-phosphate/fructose-6-phosphate phosphoketolase enzyme^10^. As an example, in *B. longum* NCC 2705, glucose, fructose, mannose, xylose, ribose and galactose were all shown to be catabolized via the “bifid-shunt”^12^.

Since carbohydrate metabolism is probably their predominant source of ATP, adequate control of carbon import and metabolic flux is important in the physiology of *Bifidobacterium* species^13^. This is reflected by the fact that bifidobacterial genomes contain a relatively large number of genes that encode carbohydrate binding Lac-I type transcription regulators, which are often predicted to regulate genes located in their close vicinity^14^. As an example, the ROK-family regulator AraU and the LacI-family regulator AraQ were predicted to control adjacent operons encoding putative arabinose transporters and catabolic enzymes^15^. However, in *B. breve* UCC 2003 AraQ, together with another Lac-I type regulator MalR1, were demonstrated to control a large set of genes that are spread over the genome including not only genes involved in uptake and metabolism of various carbohydrates, but also genes involved in the ”bifid-shunt” as well as several transcription regulators^16^. We recently demonstrated that the production of serine protease inhibitor (serpin) of *B. longum* NCC 2705, a key bifidobacterial effector molecule, is tightly controlled by carbohydrates and established that the presence of glucose in the growth medium inhibits its production^17^. These results suggest that the canonical process known as glucose-catabolite repression is important in controlling carbohydrate utilization by bifidobacteria, although the underlying molecular mechanism and involved regulatory factors remain to be deciphered. Lac-I type regulatory proteins represent the closest homologues (24–32% identity) to the catabolite control protein A (CcpA) implicated in the glucose-catabolite repression in many Gram-positive bacteria genes^18^. Of note, *B. longum* NCC 2705 harbors 16 genes encoding Lac-I type regulators according to the RegPrecise database^19^.

The poor genetic accessibility of bifidobacteria has hampered the unambiguous analysis of the mechanisms underlying carbohydrate regulation in members of this genus. Progress has been made over the past decades, especially in improving electroporation protocols^20^ and elaborated solutions were developed to enable genetic modifications of recalcitrant *Bifidobacterium* strains^21^. Nevertheless, most available plasmid vectors have a relatively narrow replication host-range, with pMDY23 being a notable exception as it could replicate in nine different species and subspecies^21^. Altogether, validated gene-expression and promoter-reporter systems remain relatively scarce for this genus^21,22^. To date, *Bifidobacterium* promoter activity studies mainly used enzyme reporters such as the β-glucuronidase encoded by the *gusA* gene^22–24^. Over the past decades, fluorescent protein reporters have been extensively employed to obtain high-throughput and single cell resolution expression information in both Gram-negative and Gram-positive bacteria^25–27^, including several lactic acid bacteria^28,29^. The Cyan (CFP), green (GFP), yellow (YFP) and red (mCherry) fluorescent proteins that all require oxygen for their maturation as well as the anaerobic maturing cyan-blue Evoglow-Pp1 fluorescent protein have been functionally expressed in different bifidobacterial species^30–32^, but were mainly used up to now to track the presence of labelled cells in different environments^31–33^ rather than studying promoter regulation.

In the present study, we first describe the selection of an appropriate fluorescent reporter protein and its validation using *B. longum* NCC 2705 promoters of different strengths that were previously characterized using the β-glucoronidase (*gusA*) based reporter^24,33^. We then demonstrated that mCherry can be used to decipher the activity and activation patterns of two promoters (P_*Bl1518*_ and P_*Bl1694*_) controlled by the AraQ and AraU transcriptional regulators, which were predicted to be implicated in arabinose utilization but also proposed to act as global transcriptional regulators in bifidobacteria. Taking advantage of the single cell resolution of fluorescent protein-based reporters we further established that when combined with flow cytometry, mCherry enables the measurement of (sub-)population adaptation during carbohydrate substrate transition and underpins the distinction between sequential utilization and co-consumption of different sugars present in the medium. Altogether, our data demonstrate the value of fluorescent protein reporters in shedding light on the regulation of carbohydrate metabolism in *B. longum* NCC 2705, providing a detailed resolution of the way this strain behaves in a complex substrate environment.

## Results

### Selection and validation of fluorescence-based reporters for promoter activity measurement in B. longum NCC 2705

We first aimed at selecting a reporter displaying an optimal fluorescence signal. For that purpose, the genes encoding fluorescent proteins that either require oxygen for their activity (GFP, CFP, YFP, mCherry) or are fluorescent under anaerobic conditions (Pp1, Bs1, Bs2) were cloned and expressed in *B. longum* NCC 2705 under control of the strong P_*gap*_ promoter that drives the expression of glyceraldehyde 3-phosphate dehydrogenase in *B. bifidum* S17. Recombinant *B. longum* NCC 2705 producing the GFP & YFP proteins emitted a fluorescent signal that was distinguishable from the autofluorescence detected in the wild-type cells, while the specific detection of CFP in the corresponding producing strain was hampered by strong autofluorescence displayed by NCC 2705 (Fig. S2). The strain harboring the pVG-mCherry (Table S1) produced the strongest relative fluorescence signal, which in part was attributable to the lack of autofluorescence produced by the wild-type cells at the corresponding excitation and emission wavelengths (Fig. S2). Amongst fluorescent proteins that do not require oxygen exposure for their maturation, Pp1 expressed in *B. longum* NCC 2705 under control of the P_*gap*_ promoter (pSDU03) generated the highest intensity signal relative to the mild autofluorescence detected for the wild-type cells (Fig. S3).

**Figure 1:**
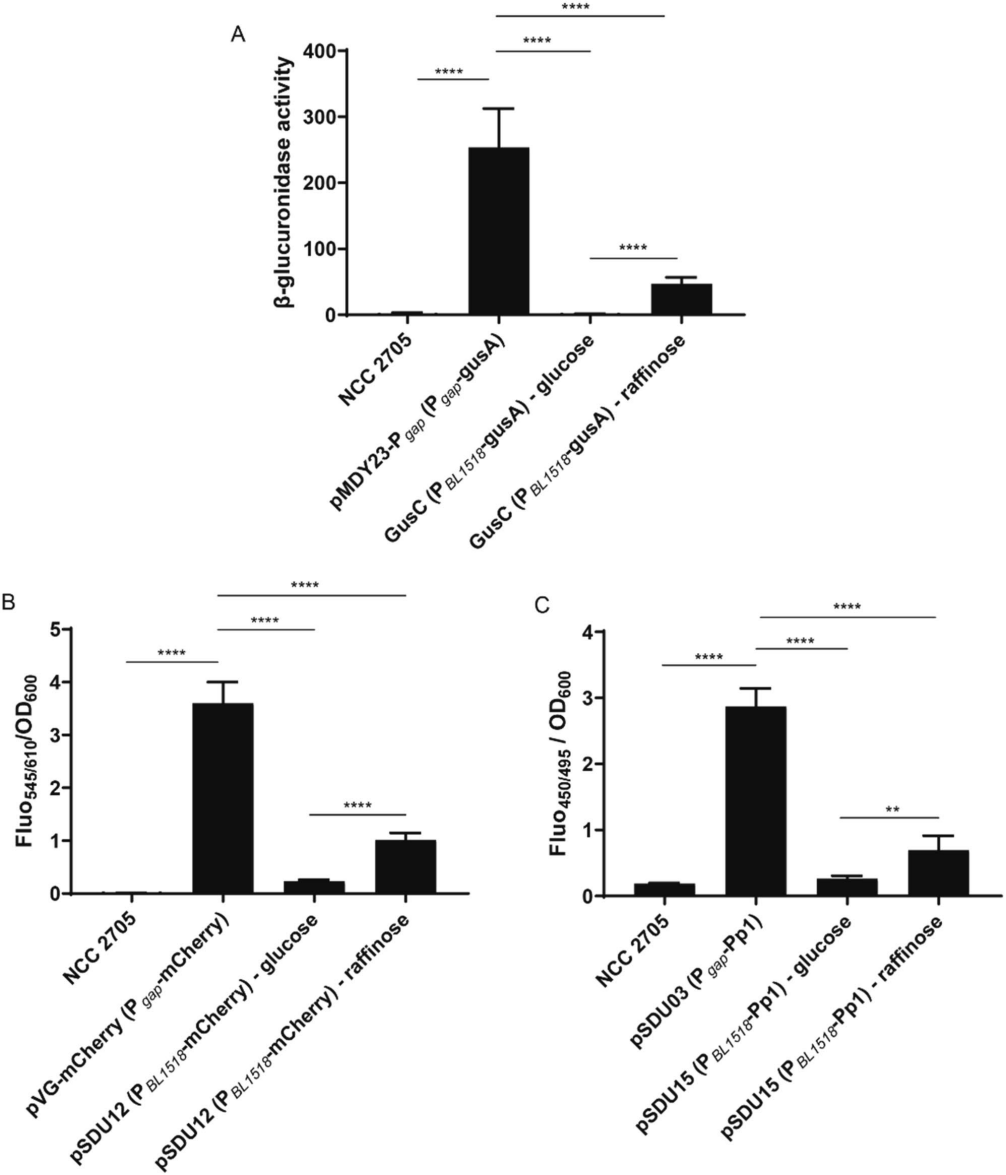
*B. longum* NCC 2705 promoter activities measured using different reporter systems in mid-exponentially grown cells. Background activity/fluorescence of the wildtype and recombinant strains harboring reporter genes under the control of P_*gap*_ (grown on glucose) or P_*BL1518*_ (grown on glucose or raffinose) was measured through β-glucoronidase (**A**), fluorescence emitted by mCherry (**B**) and fluorescence emitted by the anaerobic cyan-blue Pp1 (**C**). Values represent average of biological triplicates. Significant differences were calculated using one-way ANOVA, followed by a Sidak’s multiple comparison test (**P-value < 0.01; ****P-value < 0.0001).

Next, to validate that fluorescent proteins can be used to monitor different levels of promoter activity in *B. longum* NCC 2705, the genes encoding the best candidate fluorescent proteins (i.e., mCherry and Pp1) were cloned under control of the raffinose inducible P_*Bl1518*_ promoter^24,33^. This promoter and the P_*gap*_ promoter were previously characterized using the *gusA* reporter system and thereby provide a congruent set of promoter-reporter constructs for comparative analysis (Table S1)^24,33^. Initial experiments using *B. longum* NCC 2705 derivatives that harbor the previously described *gusA* reporter constructs confirmed previously reported results^24,33^, including the lack of activity of P_*Bl1518*_ in cells grown on glucose, the activation of this promoter during growth on raffinose (35-fold induced GusA expression), as well as the highest level of GusA activity detected in cells harboring the P_*gap*_-*gusA* reporter construct (> fivefold higher activity compared to raffinose grown P_*Bl1518*_-*gusA* harboring strain) (Fig. 1A). The fluorescence levels measured for *B. longum* NCC 2705 cultures harboring the mCherry or Pp1 genes under the control of the P_*gap*_ and P_*Bl1518*_ promoters principally recapitulated the results obtained with the corresponding *gusA* reporter constructs (Fig. 1B,C). P_*gap*_ was confirmed to be the most active promoter generating the highest fluorescence levels with either the mCherry or Pp1 reporter constructs (Fig. 1B,C). Moreover, the induction of P_*Bl1518*_ during growth on raffinose compared to growth on glucose could be confirmed for both fluorescent protein reporters, albeit with a substantially lower fold-induction (3.5- and threefold for the mCherry and Pp1 constructs, respectively) compared to the β-glucoronidase reporter (Fig. 1). This difference appeared to be attributable to background fluorescence levels detected in glucose grown cultures harboring the mCherry or Pp1 reporters cloned under control of the P_*Bl1518*_, whereas the β-glucoronidase expression was barely detectable under these conditions. In addition, the wildtype strain displayed a relatively high autofluorescence at the Pp1 excitation and emission wavelengths, which may compromise accurate activity measurements of promoters with low levels of activity. Notably, similar high autofluorescence signals at the wavelengths used for Pp1 activity measurement were also detected in *B. longum* subsp. *infantis*, *B. breve*, and *B. animalis* subsp. *lactis*, which may limit the use of this fluorescent protein reporter in *Bifidobacterium* spp. (Fig. S3). Importantly, barely detectable background autofluorescence levels were observed for the mCherry excitation and emission wavelengths in *B. longum* NCC 2705 (Fig. 1) or the other *Bifidobacterium* spp. (Fig. S3). Background autofluorescence levels seriously impact the assessment of promoter activity, which is obvious when comparing the P_*gap*_ activity assessed by the mCherry and Pp1 reporters relative to the autofluorescence background in *B. longum* NCC 2705 (600- and 16-fold, respectively; Fig. 1) and *B. breve* ATCC 15,700 (54- and fivefold, respectively; Fig. S4).

Taken together, these results demonstrate that both mCherry and Pp1 can be used as promoter-reporter proteins in *B. longum* NCC 2705 and other *Bifidobacterium* spp.. Despite the fact that mCherry requires oxygen for its maturation, which limits its use to post-cultivation activity determinations, our results indicate that it provides a superior sensitivity and dynamic range as compared to Pp1. For these reasons, we decided to use mCherry as the fluorescent protein reporter of choice in our subsequent experiments (see below).

### Carbohydrate regulation of AraQ and AraU targeted promoters, P_BL1359_ and P_BL1694_

**Figure 2:**
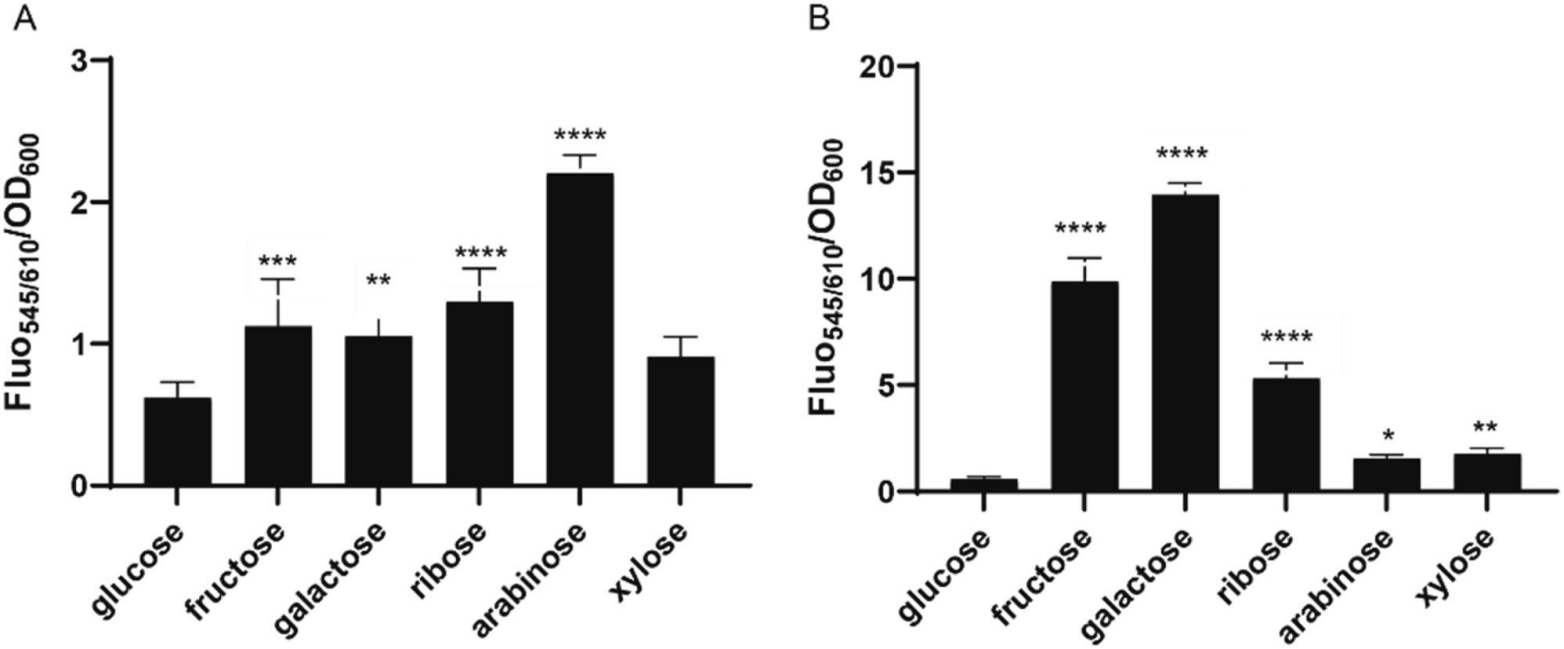
*B. longum* NCC 2705 promoter activities of mid-exponentially grown cells on 1% different hexoses and pentoses. Promoter activity is measured as the fluorescence emitted by the mCherry protein, normalized by the cell density (OD 600 nm). Fluorescence emitted by the cells harboring the pSDU30 (P_*BL1359*_-mCherry) or the pSDU32 (P_*BL1694*_-mCherry) construct is depicted in panels (**A**) and (**B**), respectively. Values represent average of biological duplicates and technical triplicates. Significant differences were calculated using one-way ANOVA, followed by a Sidak’s multiple comparison test (*P-value < 0.05; **P-value < 0.01; ***P-value < 0.001; ****P-value < 0.0001).

The mCherry-based reporter system was used to study carbohydrate mediated regulation of promoter activity in *B. longum* NCC 2705 using two exemplary promoters. The first promoter sequence we selected is located upstream of the aldose-1-epimerase encoding gene (*galM; BL1359*) (Fig. S5A). This promoter has been predicted to be regulated by AraQ (*BL0275*)^19^, an arabinose binding Lac-I type regulator protein that is conserved in *Bifidobacterium* species^15^. AraQ is not only implicated in the regulation of arabinose metabolism but has also been reported to control genome-wide regulation of gene expression in bifidobacteria^14,16^, including the closely located glyceraldehyde-3-phosphate dehydrogenase (*gap*) gene (Fig. S5A). Glucose grown cells harboring the P_*BL1359*_-mCherry reporter construct (pSDU30) did produce a 100-fold higher fluorescence level compared to the autofluorescence levels detected in untransformed wildtype cells (data not shown). The P_*BL1359*_ promoter was shown to be induced to its highest level when the strain was cultured on arabinose (3.6 fold induction compared to glucose grown cells) (Fig. 2A). It was also activated in cells growing on fructose, galactose and ribose (1.8, 1.75 and 2.2 fold induction, respectively), while no significatively higher promoter activity was observed in cells growing on xylose (Fig. 2A).

The second promoter region that was selected (P_*BL1694*_) is located upstream of the *BL1694-BL1696* operon, which is annotated to encode a multiple sugar ABC transporter^9^ (Fig. S5B). Homologues of this transporter, annotated as AraFHG, were shown to be present in several Actinobacteria families and were proposed to be involved in arabinose import^15^. AraFGH encoding genes are widely distributed in *Bifidobacteriaceae* genomes and are predicted to be regulated by the adjacent but divergently transcribedA*araU* gene (*BL1693*) that encodes a ROK-family transcriptional regulator^15,19^. It is worth to note that the region we cloned is predicted to contains two divergent AraU binding sites, controlling the expression of *BL1693*-*1692* (P_*BL1693*_) and *BL1693*-*1695* (P_*BL1694*_), respectively (Fig. S5B) and that our cloning strategy specifically aimed at deciphering the activity of P_*BL1694*_. While glucose grown cells harboring the P_*BL1694*_-mCherry construct (pSDU32) exhibited a fluorescence level similar to that observed in glucose-grown cells harboring pSDU30 (P_*BL1359*_-mCherry), P_*BL1694*_ activity was strongly induced when cells were grown on fructose and galactose (20- and 28-fold relative to the glucose expression level, respectively). Notably, the *BL1694* promoter activity was also stimulated during growth on the pentoses ribose and xylose, and during growth on arabinose albeit to a lower level compared to galactose or fructose (Fig. 2B).

These results establish that both *P*_*BL1359*_ and *P*_*BL1694*_ are controlled by carbohydrates, as they have a relatively low activity on glucose and are activated by growth on more than a single carbohydrate.

### Population behavior of B. longum NCC 2705 switching from glucose to arabinose or galactose

We further investigated the role of glucose in the regulation of *P*_*BL1359*_ and *P*_*BL1694*_. Moreover, we exploited the capacity of flow cytometry to detect single cell-fluorescence levels to assess community behavior during transition from one carbon source to another, e.g., from glucose to either arabinose or galactose.

**Figure 3:**
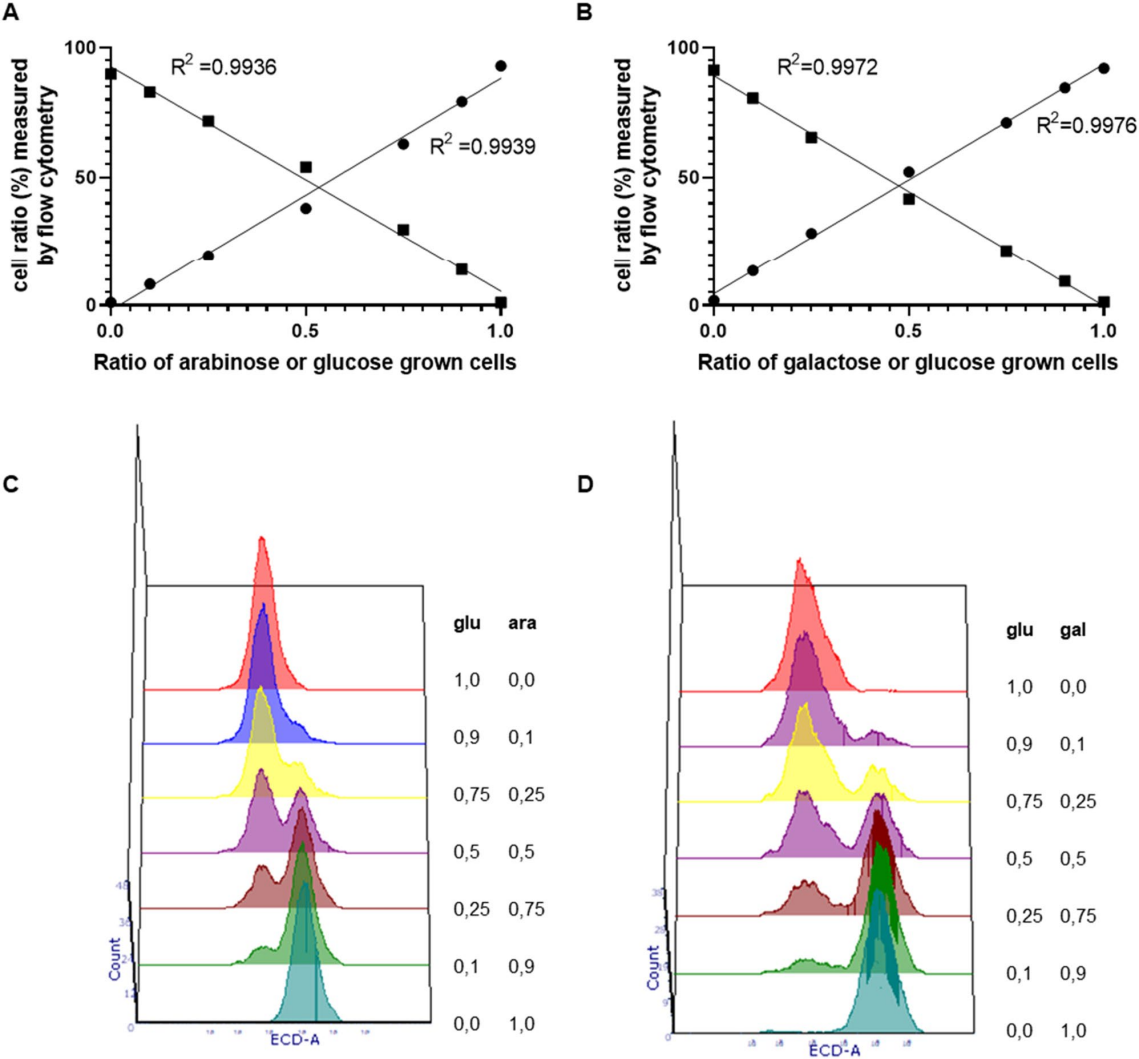
Flow cytometry scatter plots of stationary grown *B. longum* NCC 2705 cells harboring the pSDU30 (P_*BL1359*_-mCherry) (panel **A** and **C**) and the pSDU32 (P_*BL1694*_–mCherry) (panel **B** and **D**) constructs. Populations were mixed at different ratios (A & C [glucose:arabinose cells], **B** and **D** [glucose:galactose cells]) of 1.00:0.00, 0.90:0.10, 0.75:0.25, 0.50:0.50, 0.25:0.75, 0.10:0.90 and 0.00:1.00. Quantification of high (circles) and low (squares) fluorescent populations in the different mixes was performed by flow cytometry. Lines represent linear regression for each population (Panels **A** and **B**).

We first confirmed that the mCherry reporter system we developed allows the detection and quantification of bacterial sub-populations. To this end, we performed a validation experiment with mixtures at different ratios of stationary phase cultures of *B. longum* NCC 2705 harboring pSDU30 (P_*BL1359*_-mCherry) and pSDU32 (P_*BL1694*_ – mCherry) grown on glucose or arabinose, and on glucose or galactose, respectively. The different mixtures were analyzed by flow cytometry, revealing an almost perfect separation and quantification of the high (arabinose or galactose grown, respectively) and low (the glucose grown counterparts) fluorescent populations present in the mixtures (Fig. 3). This result demonstrates that the mCherry reporter system allows to distinguish sub-populations of cells growing on different sugars with very high accuracy, but also confirmed the bi-modal activation of P_*BL1359*_ and P_*BL1694*_, both promoters being mostly repressed on glucose and activated on arabinose or galactose, respectively.

**Figure 4:**
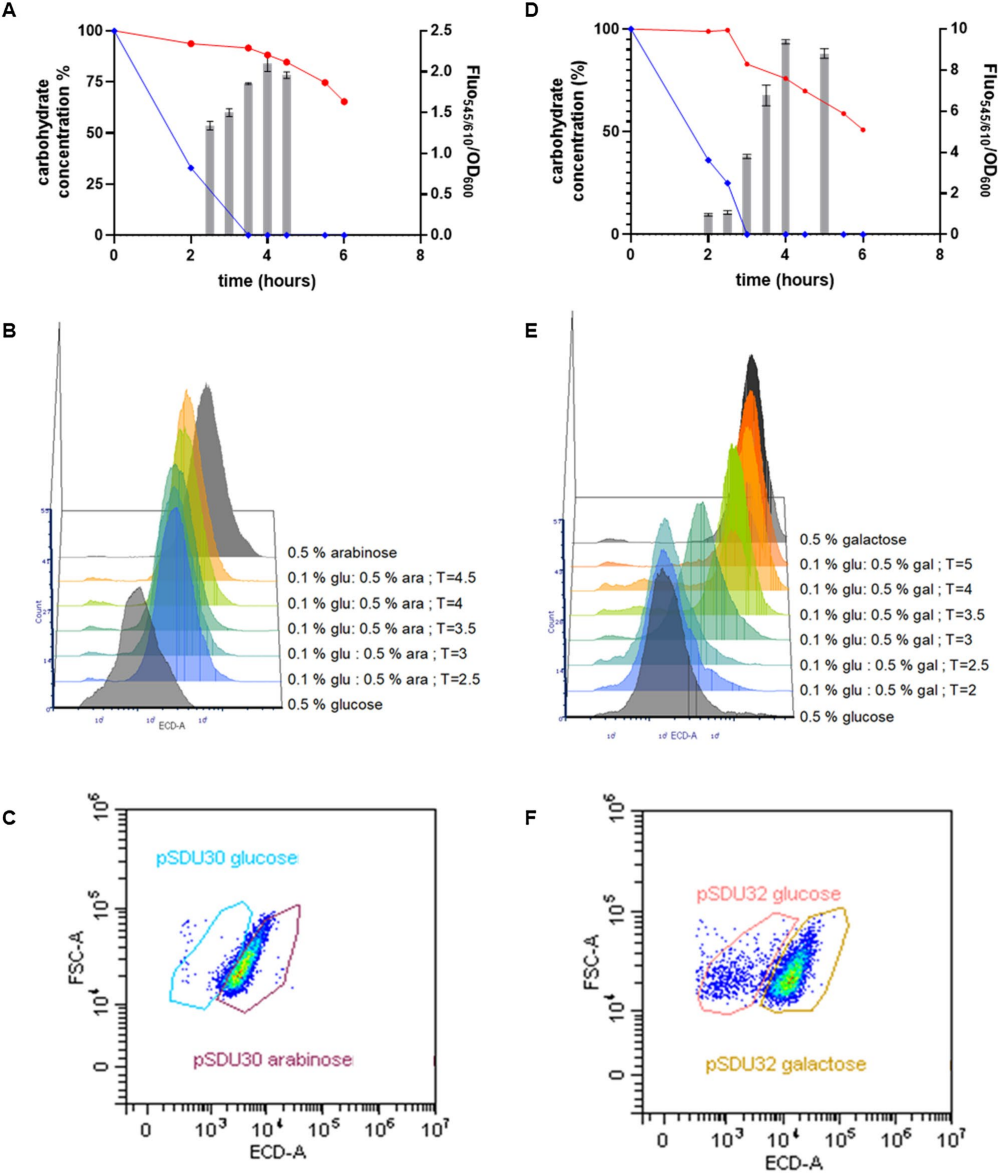
Behavior of *B. longum* NCC 2705 harboring the pSDU30 (P_*BL1359*_-mCherry) plasmid cultured on 0.1% glucose and 0.5% arabinose (panels **A**, **B** and **C**) or harboring the pSDU32 (P_*BL1694*_-mCherry) plasmid cultured on 0.1% glucose and 0.5% galactose (panels **D**, **E** and **F**). Above panels show the evolution of the fluorescence signal emitted around the glucose depletion timepoints (grey bars) and the carbohydrate consumption patterns (panel **A**: glucose (blue) and arabinose (red); panel (**D**) glucose (blue) and galactose (red)). Middle panels present the evolution of the populations measured by flow-cytometry around the glucose depletion timepoint (colored), comparing them to glucose and arabinose (grey) (panel **B**) or glucose and galactose grown cultures (panel **E**). Below panels represent the population distribution 1 h after glucose depletion of both pSDU30 harboring cells (T = 4.5, panel **C**) and pSDU32 harboring cells (T = 4, panel **F**).

We next analyzed sugar utilization and population adaptation of the recombinant *B. longum* NCC 2705 strains containing pSDU30 (P_*BL1359*_-mCherry) or pSDU32 (P_*BL1694*_-mCherry) during the transition from glucose to arabinose or from glucose to galactose, respectively. To this end, the NCC 2705 strains harboring pSDU30 or pSDU32 were grown on MRSc-C media supplemented with 0.1% glucose and 0.5% arabinose, or 0.1% glucose and 0.5% galactose, respectively. During growth, the consumption of the available sugars was followed while in parallel, single-cell fluorescence levels were measured in the population by flow cytometry. The results clearly demonstrated that pSDU30 harboring *B. longum* NCC 2705 consumed glucose and arabinose simultaneously during the initial hours of growth, which was supported by relatively high average levels of fluorescence in the culture that reflect substantial activity of the P_*BL1359*_ promoter as a proxy for expression of the arabinose utilization genes (Fig. 4A,B). After approximately 4 h, glucose was depleted from the medium, which coincided with a modest but significant increase in average fluorescence in the culture, indicating that upon glucose depletion the activity of the P_*BL1359*_ promoter is further enhanced. This is in agreement with the observed acceleration (4.6 ×) of the rate of arabinose consumption after glucose was depleted (Fig. 4A), indicating that despite glucose being exhausted, cells are alive and metabolically active. Analogously, the fluorescence levels measured for single cells in the culture increased towards the level observed in cells grown on arabinose alone only after glucose was depleted (Fig. 4B,C). These results show that the presence of arabinose in the medium alleviates the observed glucose-suppression of P_*BL1359*_ activity, but that the activity of this promoter can still be further enhanced when glucose is depleted, indicating that the presence of glucose still mediates a partial level of repression in media that also contain the arabinose.

These results strikingly contrasted with those obtained for the strain harboring pSDU32, containing the P_*BL1694*_-mCherry reporter. During initial growth the cells appeared to exclusively utilize glucose, which is in agreement with low average P_*BL1694*_ activity in the culture. Following the depletion of glucose from the medium (after approximately 3 h), the strain remained alive and started to actively utilize galactose for growth which coincided with activation of P_*BL1694*_ promoter, reflected by the sharp increase of the average fluorescence levels in the culture (Fig. 4D). This typical diauxic growth characteristic on is in agreement with the single cell analysis, which showed that initial fluorescence levels were similar to those observed in cultures grown on glucose alone and once glucose was depleted single cell fluorescence levels sharply increased to a level that is also observed in cultures grown on galactose as the sole carbon source (Fig. 4E). Notably, during the transition towards galactose utilization, transient sub-populations of cells growing on either glucose or galactose were observed (Fig. 4F), eventually leading to full activation of the P_*BL1694*_ promoter in all cells of the culture (Fig. 4E). These results demonstrate a community-wide adaptation to the utilization of galactose.

Taken together, these results strongly suggest that in *B. longum* glucose mediated catabolite repression is exerted solely on specific sugars such as galactose while not affecting other sugars such as arabinose. The physiological consequences of this differential glucose regulation led to sequential consumption of glucose and galactose but to co-consumption of glucose and arabinose.

## Discussion

Bifidobacteria are saccharolytic bacteria, and their capacity to metabolize a large range of carbohydrate renders them particularly adapted to the complex gut ecosystem. Detailed study of the fine regulation mechanisms controlling their carbohydrate metabolism requires single cell level molecular tools. Fluorescent protein-based reporters represent therefore an interesting alternative to the commonly used glycan-hydrolase based reporter systems. Fluorescent proteins have been previously expressed in bifidobacteria to enable their real-time detection in in vitro or in vivo systems^32,33^, but to the best of our knowledge, they have not yet been applied in this genus to study the detailed carbohydrate regulation of selected promoters.

In the present study, we evaluated whether fluorescent reporter-proteins that do or do not require oxygen to mature (mCherry and Evoglow-Pp1, respectively) could be used to measure the activity of specific promoters with better precision than the commonly used GusA reporter. Fluorescent proteins that do not require aerobic conditions for their maturation would be most compatible with the anaerobic lifestyle of bifidobacteria. Out of the 3 tested anaerobic fluorescent proteins, Pp1 was identified as the most promising candidate. However, a major drawback of the Pp1 reporter system is that wild-type bifidobacterial strains, including *B. longum* NCC 2705, exhibit a relatively high level of autofluorescence at the excitation and emission wavelengths of this reporter, which decreases the sensitivity and dynamic range of the Pp1-based promoter-reporter system in bifidobacterial strains. The mCherry reporter displayed the most attractive characteristics as it led to a high intensity signal combined with very low or even barely detectable autofluorescence in *B. longum* NCC 2705 as well as other *Bifidobacterium* spp. The requirement for oxygen exposure for mCherry maturation restricts the use of this reporter protein to post-cultivation analysis, which in general would be considered a disadvantage. Nevertheless, by including an appropriate incubation period in the presence of oxygen, we successfully used this protein as reporter to accurately measure promoter activities in anaerobically grown *B. longum* NCC 2705. The design of an anaerobic fluorescent marker with excitation and emission wavelengths similar to those of mCherry would facilitate in situ promoter activity evaluation in growing *B. longum* NCC 2705. Interestingly, the biarsenical-tetracysteine tag system was successfully employed for imaging and detection of the anaerobic gut bacterium *Bacteroides thetaiotaomicron*^34^. This system can covalently react with the green fluorescein (FlAsH) or the red resofurin (ReAsH)^35^ and may thereby represent an interesting alternative to mCherry, although the previously reported non-specific ligand binding could present challenges for the use of this system as a promoter-reporter^36^.

Nevertheless, we took advantage of the single cell resolution of the mCherry based reporter system to decipher the carbohydrate regulation patterns of the P_*BL1359*_ and P_*BL1694*_ promoters predicted to be respectively regulated by AraQ and AraU, two regulatory proteins implicated in arabinose utilization^14–16^. The Lac-I type regulator AraQ was additionally proposed to act as global transcriptional regulator in bifidobacteria^15,16^, thus potentially representing a functional homologue of the typical Gram-positive CcpA which appears to be absent in *Bifidobacterium* strains. The mechanism by which AraQ may exert its global regulatory role is however likely different to that of the negative regulator CcpA, as AraQ acts both as an activator and a repressor^15,16^. We demonstrated that P_*BL1359*_, located upstream of the aldose-1-epimerase encoding gene (BL1359; *galM*) was mildly activated when *B. longum* NCC 2705 was grown on various sugars, including fructose, galactose and ribose, relative to the activity level observed when cells were grown on glucose. These results are in line with the proposed glucose mediated catabolite repression that was recently reported for the regulation of serpin expression in the same strain^17^. Furthermore, the enolase encoding gene *BL1022* predicted to be regulated by AraQ was shown to be differentially expressed in glucose, xylose and galactose, further supporting the role of AraQ in catabolite repression in *B. longum* NCC 2705^12^. Notably, the P_*BL1359*_ activity was most prominently induced in cells growing on arabinose as a carbon source, which is in agreement with the prediction that this promoter is regulated by AraQ^19^. The mCherry based reporter allowed to demonstrate that the arabinose-mediated induction of the P_*BL1359*_ promoter was to a large extend also occurring in the presence of glucose in the whole cell population, which correlates with the observed co-consumption of these two sugars by *B. longum* NCC 2705. These findings imply that the proposed role of AraQ in glucose-mediated catabolite repression is partially alleviated in presence of arabinose, an intriguing observation that warrants further investigation.

The carbon source regulation characteristics of P_*BL1359*_ were fundamentally different from those observed for P_*BL1694*_. The latter promoter is located upstream of an operon that is predicted to encode a multi sugar ABC transport system previously shown to be induced when cells are grown on short-chain fructo-oligosaccharides^9^. This operon was recently annotated as *araFDH* and proposed to play a role in arabinose transport. The ROK family repressor protein AraU encoded by the gene *BL1693* was hypothesized to control its own expression, but also that of the neighboring *araFDH* operon through regulation of the P_*BL1694*_ promoter^15,19^. Our data clearly establish that P_*BL1694*_ was activated during growth on several hexoses like fructose and galactose in comparison to growth on glucose. It was however barely activated by growth on xylose and arabinose, which does not support the previously suggested role of the *araFDH* encoded ABC transport system in arabinose transport^15^. In addition, using the mCherry reporter system, we could demonstrate that the galactose mediated activation of P_*BL1694*_ is inhibited in the entire cell population in presence of glucose, which is in agreement with the observation that galactose is not consumed when glucose is also present. Based on these observations, we propose that the ROK family regulator AraU and its downstream ABC transporter (*araFDH*) play a role in galactose (and potentially fructose) transport in *B. longum* NCC 2705. The activation pattern of P_*BL1694*_ shares several similarities to the arabinose inducible P_*BAD*_ promoter that has been used to construct widely used sets of inducible expression vectors in *E. coli*^37,38^. Therefore, the P_*BL1694*_ promoter could represent an interesting option for the construction of a galactose-inducible gene expression system for *B. longum* NCC 2705 and possibly other bifidobacteria in which the regulation of the AraFDH transporter is conserved.

Taken together, we demonstrated that the mCherry promoter-reporter system could recapitulate the relative activity levels of promoters previously characterized using the more traditional β-glucoronidase reporter^24^. In addition, the mCherry reporter allowed to study promoter activation at single cell level, which allowed us to unravel the population wide carbohydrate-activation profiles of two promoters that were previously suggested to play an important role in arabinose consumption (as well as other sugars) by *B. longum* NCC 2705^15^. We established that *B. longum* NCC 2705 behaves differently depending on the carbohydrates it is exposed to, displaying distinct transition phenotypes when switching from glucose to arabinose or to galactose, where glucose and arabinose appeared to be co-metabolized, glucose and galactose were used sequentially. Interestingly, the time required to reach glucose depletion was extended when this sugar was co-metabolized with arabinose, suggesting that co-metabolization reduces the utilization rate for both sugars. Finally, during the transition from glucose to galactose utilization we observed the transient coexistence of two sub-populations that probably grow on either glucose or galactose. Nevertheless, in *B. longum* NCC 2705 the whole population shifted finally to galactose utilization. This population-wide adaptation in NCC 2705 contrasts with previous *L. lact*is observations where only a specialized sub-population is at the origin of the glucose to galactose metabolic shift^39^.

Our results illustrate how fluorescent protein promoter-reporters can be used to shed light on the complex regulation of carbohydrate metabolism in *B. longum* NCC 2705. Especially when combined with flow cytometry, these reporters allow the study of (sub-)population adaptation behavior in *B. longum* NCC 2705 residing and growing in environments containing more than a single carbohydrate substrate. In the dynamic environment of the intestinal tract it is highly plausible that bifidobacteria are frequently exposed to different carbohydrate substrates simultaneously, which underpins the relevance of approaches like the one we followed here to unravel how these bacteria coordinate their metabolic versatility and adaptation under such conditions.

## Methods

### Plasmid constructs

Intensity of different aerobic and anaerobic fluorescent proteins was tested in *B. longum* NCC 2705. Plasmid pMDY23^24^ and its derivatives encoding GFP (pVG-GFP), YFP (pVG-YFP), CFP (pVG-CFP) and mCherry (pVG-mCherry) under the control of the strong *B. bifidum* P_*gap*_ promoter were generously provided by Dr. Christian Riedel (Ulm University, Germany) (Table S1)^33^. The sequences coding for three different cyan-green anaerobic fluorescent proteins (Bs1, Bs2 and Pp1) were obtained from Evocatal GmbH (Düsseldorf, Germany). Codon optimized (for expression in bifidobacteria) variants of these genes were synthesized (Thermo Fisher Scientific/Geneart GmbH, Regensburg, Germany) and cloned under the control of the P_*gap*_ promoter by replacing the mCherry encoding region (encompassed between XhoI and SacII restriction sites) of pVG-mCherry, yielding pSDU01 (P_*gap*_-Bs1), pSDU02 (P_*gap*_-Bs2) and pSDU03 (P_*gap*_-Pp1) (Table S1).

The Pp1 and mCherry fluorescent proteins were subcloned under control of the previously described raffinose-inducible promoter preceding *BL1518* in *B. longum* NCC 2705 (P_*BL1518*_; genome coordinates 1,692,388 to 1,692,679)^24^. The P_*BL1518*_ fragments harboring different restriction sites at each end were synthesized and cloned in front of mCherry and Pp1 encoding genes (see pVG-mCherry plasmid map, Fig. S1) (Thermo Fisher Scientific/Geneart GmbH). For mCherry this was achieved by replacing the BglII-MscI P_*gap*_ promoter fragment in pVG-mCherry by the similarly digested P_*BL1518*_ fragment, yielding pSDU12 (P_*BL1518*_-mCherry). Similarly, P_*BL1518*_ was used to replace the P_*gap*_ region in pSDU03 using the BglII-BclI cloning sites, generating pSDU15 (P_*BL1518*_-Pp1) (Thermo Fisher Scientific/Geneart GmbH) (Table S1). Two plasmids encoding β-glucoronidase under the control of the P_*gap*_ (P_*gap*_-*gusA*; pMDY23^33^) and the raffinose inducible P_*BL1518*_ (P_*BL1518*_-*gusA*; pGUSC^24^) were used as controls (Table S1).

The promoter preceding the aldose-1-epimerase encoding gene (P_*BL1359*_; genome coordinates 262,095 to 261,970; reversed sequence) and the promoter upfront of the *BL1694* gene encoding the proposed pentose binding protein (P_*BL1694*_; genome coordinates 2,114,397 to 2,114,649) (Fig. S5) were synthesized and subcloned in the pVG-mCherry plasmid replacing the BglII-MscI P_*gap*_ promoter fragment in pVG-mCherry, resulting in the pSDU30 (P_*BL1359*_-mCherry) and pSDU32 (P_*BL1694*_-mCherry) plasmids, respectively (Thermo Fisher Scientific/Geneart GmbH). The orientation of both sequences used for the cloning was carefully selected to decipher the P_*BL1359*_ and P_*BL1694*_ promoter activities. All plasmids carried a spectinomycin resistance gene as selective marker.

### Strain cultivation and transformation

All strains were retrieved from the Nestle Culture Collection (NCC, Nestlé Research, Lausanne, Switzerland) and were routinely cultured in MRS medium supplemented with 0.05% cysteine-HCl (MRSc), under anaerobic conditions at 37 °C, in a closed jar containing an AnaeroGen sachet (ThermoFischer Scientific GmbH). *B. longum* NCC 2705 and *B. breve* ATCC 15,700 (T) electrocompetent cells were prepared using the method previously described by Serafini et al.^20^. Strains were grown to stationary phase (overnight incubation) in fresh MRSc supplemented with 16% (w v^−1^) of short chain fructo-oligosaccharides (scFOS) (Actilight®; Beneo-Orafti, Oreye, Belgium) at 37 °C in anaerobiosis. This preculture was diluted 1:10 in 200 ml of fresh MRSc broth and 16% scFOS, and cultivated at 37 °C until an OD_600_ nm of 0.6–0.7 was reached. Cultures were cooled on ice and cells were harvested by centrifugation (2500 g for 10 min), then washed twice with 20 ml of ice-cold washing buffer (16% FOS in a 1 mM citrate buffer, pH 6.0). Finally, cells were resuspended in 800 µl (1/250 of the original culture volume) of the same ice-cold buffer. Aliquots of 80 µl were frozen in liquid nitrogen and stored at − 80 °C until use. Cells were thawed on ice and plasmid DNA (200 ng) was transformed by electroporation in a Nucleofector device and using the AA-035 protocol settled in the apparatus (RUWAG Handels AG, Bettlach, Switzerland). Electroporated cells were recovered in 1 ml of fresh MRSc and incubated for 2-3 h in anaerobiosis at 37 °C before plating. Transformant colonies were obtained after 48 h of growth at 37 °C in anaerobiosis on MRSc agar containing 200 µg ml^−1^ of spectinomycin (MRScs). Transformants were grown anaerobically overnight at 37° in MRScs and stored at -80 °C after addition of 20% glycerol (v v^−1^).

### β-glucoronidase activity measurement

Overnight anaerobically grown cultures of *B. longum* NCC 2705 containing the plasmids carrying the β-glucoronidase encoding gene (*gusA*) under the control of the different promoters were subcultured in 30 ml of fresh MRSc based medium lacking a carbon source (MRSc-C) (10 g l^−1^ of bacto proteose peptone n°3, 5 g l^−1^ bacto yeast extract, 1 g l^−1^ Tween 80 [all from Chemie Brunschwig, Buchs, Switzerland], 2 g l^−1^ di-ammonium hydrogen citrate, 5 g l^−1^ sodium acetate, 0.1 g l^−1^ magnesium sulphate, 0.05 g l^−1^ manganese sulfate, 2 g l^−1^ di-sodium phosphate, 0.5 g l^−1^ cysteine [all from Sigma-Aldrich Chemie GmbH]) to which 2% of glucose or raffinose was added. Growth experiments were performed in biological triplicates, at 37 °C under anaerobiosis and until mid-logarithmic phase (OD_600_ ~ 0.6). All media contained 200 µg ml^−1^ of spectinomycin.

Cells were harvested from mid-exponential cultures by centrifugation (2500 g for 10 min at 4 °C) and concentrated to reach 12 OD_600_ units per ml in the previously described GusA-assay buffer^33^ consisting of 50 mM Na_2_HPO_4_ (pH 7), 1 mM EDTA, 0.1% Triton X-100 and 5 mM dithiothreitol (DTT). Cells were mechanically disrupted using glass beads, for 3 times 1 min at 4 m s^−1^ using a FastPrep-24 (MP Biomedicals LLC, Irvine, USA). Cell debris were removed by centrifugation (3300 g, 5 min, 4 °C) and 100 µl of the cell lysate was transferred to 96 well plates before being mixed with 100 µl of 15 mM 4-Nitrophenyl β-D-glucuronide (PNP-GLUC; Sigma-Aldrich Chemie GmbH) dissolved in assay buffer. Plates were incubated at 37 °C and absorbance was measured at 405 nm every 5 min for 30 min in a Varioskan spectrophotometer (Thermo Fisher Scientific). Enzyme activities were expressed as nitrophenol nanomoles released per minute.

### Promoter activity measured through fluorescence level determination

Overnight cultures of *B. longum* NCC 2705 harboring the plasmids encoding fluorescent proteins under control of the different promoters were cultured as previously described in MRSc-C to which 2% of the respective sugars (glucose, raffinose, fructose, galactose, ribose, arabinose or xylose) was added. Precultures were performed in the same medium composition to preadapt the bacterial cells to the respective substrates.

To determine cyan-blue Evoglow-Bs1, Bs2 and Pp1 fluorescence levels, cells were washed twice and resuspended in 250 mM phosphate buffer at pH 7.0. The OD_600_ of the suspensions as well as fluorescence were measured in a Varioskan spectrophotometer (Thermo Fisher Scientific) without any further incubation. Excitation and emission wavelengths of 450 and 495 nm, respectively, were used for all variants.

To determine fluorescence levels of proteins requiring oxygen to mature, cells were centrifuged (3500 g for 2 min), washed twice and resuspended in 250 mM phosphate buffer at pH 7.0 containing the bacteriostatic antibiotic Chlortetracycline (Sigma-Aaldrich chemie GmbH) at 30 µg ml^−1^ to stop protein synthesis. Cells were then incubated at room temperature (in presence of oxygen) for 90 min, and OD_600_ and fluorescence levels were measured in a Varioskan spectrophotometer (Thermo Fisher Scientific). Excitation and emission wavelengths were the following: 470 and 625 nm for GFP, 436 and 480 nm for CFP, 500 and 535 nm for YFP, and 545 and 610 nm for mCherry, respectively.

### Statistical analysis

Statistical analysis and graphical representations of the different promoter activation datasets was performed using GraphPad prism v8.1.1 (GraphPad Softwares, San Diego, USA). Average and standard deviations are represented, while significance levels were determined using a one-way ANOVA, followed by Sidak’s multiple comparison tests (α = 0.05).

### Measurement of population evolution by flow-cytometry

Cells harboring the pSDU30 (P_*BL1359*_-mCherry) and pSDU32 (P_*BL1694*_-mCherry) plasmids were grown in fresh MRSc-C supplemented with 200 µg/ml spectinomycin and different carbohydrate mixtures (0.1% glucose:0.5% arabinose and 0.1% glucose:0.5% galactose, respectively). Ten (10) ml of early exponential phase cultures were aliquoted in smaller volumes (1 ml) and incubated at 37 °C in anaerobiosis for another 12 h and collected at regular intervals throughout the exponential phase. Cells were harvested by centrifugation (room temperature, 3000 g, 2 min) and resuspended in 250 mM phosphate buffer at pH 7.0 containing 30 µg ml^−1^ Chlortetracycline (Sigma-Aldrich Chemie GmbH, Buchs, Switzerland). Population distribution was evaluated by flow cytometry after 90 min of aerobic incubation at 37 °C, which corresponds to the optimal maturation time of the mCherry protein. Residual glucose levels were determined in these cultures using the MQuant kit (Sigma-Aldrich Chemie GmbH) according to the manufacturer’s protocol and carbohydrate consumption was determined by HPLC (see below).

Flow cytometry analyses were performed with a Beckman Coulter Cytoflex S (Beckman Coulter, Brea, US) equipped with four lasers. The mCherry fluorescence was measured in the ECD detector (excitation at 561 nm, emission at 610/20 nm). Results were analyzed with the Beckman-Coulter CytExpert Acquisition software version 2.3. All data were exported in FCS 3.0 format and analyzed offline with the De Novo FCSExpress software version 7.01.

### Carbohydrate consumption measured by HPLC

Glucose, galactose and arabinose levels were quantified in filtered sterile culture supernatants by High Performance Liquid Chromatography (HPLC, Agilent Technologies AG, Basel, Switzerland), using an Aminex HPX-87H columm (Bio-Rad Laboratories AG, Cressier, Switzerland). 5 µl of samples were injected, using a 5 mM H_2_SO_4_ solution as eluent, with a flow rate of 0.6 ml/min (run time 25 min). Chromatogram was obtained using a refractive index detector, from which peak areas and corresponding concentrations were calculated.

## Supplementary Information

Supplementary Information.

## Data Availability

The datasets used and/or analysed during the current study available from the corresponding author on reasonable request.
