# Supplementary material for: Using fluorescent promoter-reporters to study sugar utilization control in Bifidobacterium longum NCC 2705
Source: Sci Rep. 2022 Jun 21;12:10477. doi: 10.1038/s41598-022-14638-4 (PMC9213400; doi:10.1038/s41598-022-14638-4)
Supplement: Supplementary file 1 — Supplementary Information. [file 41598_2022_14638_MOESM1_ESM.docx]

**Supplementary Data belonging to manuscript**

Using fluorescent promoter-reporters to study sugar utilization control in *Bifidobacterium longum* NCC 2705

S. Duboux ^1,2^, J.A. Muller ^1^, F. De Franceschi ^1^, A. Mercenier ^2^ & M. Kleerebezem ^2^

Authors affiliations:

^1^ Nestlé Research, P.O. Box 44, CH-1000 Lausanne 26, Switzerland
^2^ Host Microbe Interactions group, Wageningen University, P.O. Box 338, 6700 AH Wageningen, The Netherlands

Table S 1: List of plasmids used in this work

| material | description | origin |
| --- | --- | --- |
| pVG-YFP | YFP fluorescent protein under the control of *B. bifidum* S17 P*_gap_* | Derived from pMDY23, Grimm et al. |
| pVG-CFP | CFP fluorescent protein under the control of *B. bifidum* S17 P*_gap_* | Derived from pMDY23, Grimm et al. |
| pVG-GFP | GFP fluorescent protein under the control of *B. bifidum* S17 P*_gap_* | Derived from pMDY23, Grimm et al. |
| pVG-mCherry | mCherry fluorescent protein under the control of *B. bifidum* S17 P*_gap_* | Derived from pMDY23, Grimm et al. |
| pSDU01 | Bs1 anaerobic fluorescent protein under the control of *B. bifidum* S17 P*_gap_* | Derived from pVG-mCherry, this work. |
| pSDU02 | Bs2 anaerobic fluorescent protein under the control of *B. bifidum* S17 P*_gap_* | Derived from pVG-mCherry, this work. |
| pSDU03 | Pp1 anaerobic fluorescent protein under the control of *B. bifidum* S17 P*_gap_* | Derived from pVG-mCherry, this work. |
| pMDY23-pGap | β-glucoronidase reporter enzyme under the control of *B. bifidum* S17 P*_gap_* | Derived from pMDY23, Grimm et al. |
| pGusC | β-glucoronidase reporter enzyme under the control of *B. longum* NCC 2705 P*_BL1518_* | Derived from pMDY23, Klijn et al. |
| pSDU12 | mCherry fluorescent protein under the control of *B. longum* NCC 2705 P*_BL1518_* | Derived from pVG-mCherry, this work. |
| pSDU15 | Pp1 anaerobic fluorescent protein under the control of *B. longum* NCC 2705 P*_BL1518_* | Derived from pSDU03, this work. |
| pSDU30 | mCherry fluorescent protein under the control of *B. longum* NCC 2705 P*_BL1359_* | Derived from pVG-mCherry, this work |
| pSDU32 | mCherry fluorescent protein under the control of *B. longum* NCC 2705 P*_BL1694_* | Derived from pVG-mCherry, this work |

Figure S 1: Map of the pVG-mCherry plasmid, highlighting the restriction sites used for cloning in this work and containing the following elements: pMB1 replicon for replication in *E. coli* (black region); *repA* and *repB* for replication in bifidobacteria and the spectinomycin resistance gene (black arrows); P_gap_ promoter (red region); genes encoding the fluorescent proteins mCherry (red arrow).

Figure S 2: Fluorescence signals of MRSc stationary grown wildtype B. longum NCC 2705 and its recombinant derivatives harboring pVG-GFP (A), pVG-CFP (B), pVG-YFP (C) and pVG-mCherry (D) measured at the respective excitation and emission wavelengths of each individual reporter proteins. Data represent averages and standard deviations of biological triplicates and were determined using technical duplicates. P-values were calculated using one-way ANOVA, followed by a Sidak’s multiple comparison test. **** p-value < 0.0001 as compared to control (wildtype).

Figure S 3: Fluorescence signal of stationary grown wildtype B. longum NCC 2705 and its pSDU01, pSDU02 or pSDU03 harboring transformants measured at excitation and emission wavelengths of 450 and 495 nm, respectively. Values represent average of biological triplicates. Significant differences between the background fluorescence of the wild-type and recombinant strains were calculated using one-way ANOVA, followed by a Sidak’s multiple comparison test (**** p-value < 0.0001).


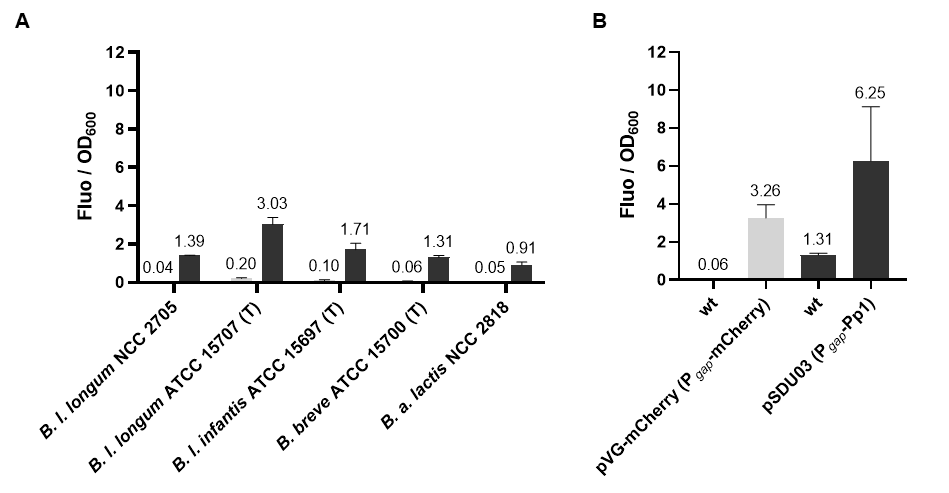


Figure S 4: Comparison of fluorescence emitted by mCherry and Pp1 reporter proteins in different bifidobacterial hosts. Fluorescence signal of MRSc stationary grown wildtype B. longum NCC 2705, B. longum ATCC 15707 (T), B. longum subsp. infantis ATCC 15697 (T), B. breve ATCC 15700 (T) and B. animalis subsp. lactis NCC 2818 measured at excitation and emission wavelengths of 545 and 610 nm (light grey bars), as well as 450 and 495 nm (dark grey bars), respectively (panel A). Fluorescence signal emitted at the same wavelengths by wildtype and P_gap_ recombinant derivatives of B .breve ATCC 15700 (T) (panel B). Values represent average of biological duplicates.


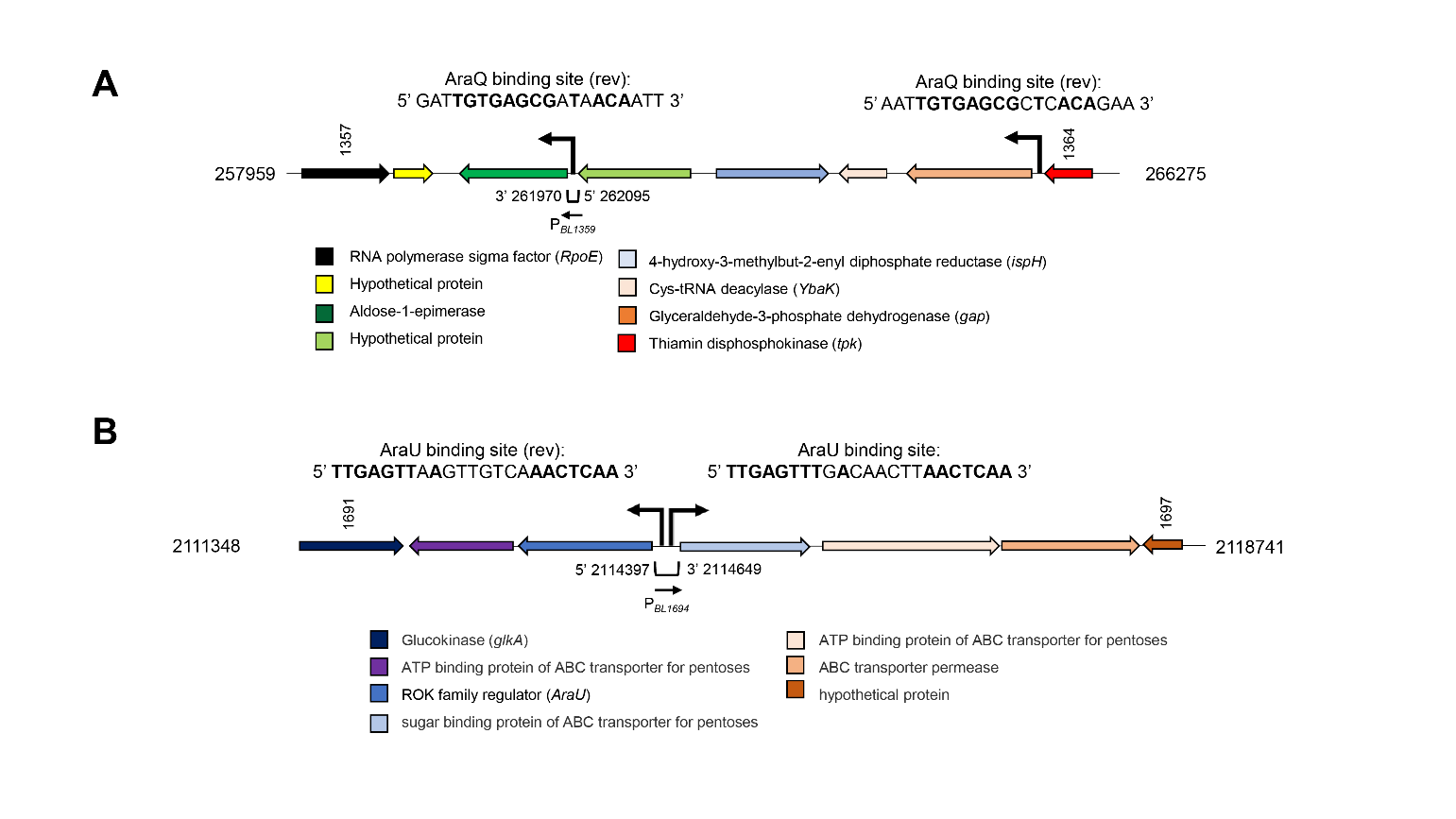


Figure S 5: Operon organization of the two regions encompassing AraQ (Panel A) and AraU (Panel B) targeted cloned promoters (P_BL1359_ & P_BL1694_). Genes and orientations are indicated by colored arrows. The promoter regions and their directions are indicated in respective intergenic regions together with the predicted RegPrecise binding sites. Conserved binding motifs are highlighted in bold.
